# Loss of threonyl-tRNA synthetase-like protein Tarsl2 has little impact on protein synthesis but affects mouse development

**DOI:** 10.1016/j.jbc.2023.104704

**Published:** 2023-04-12

**Authors:** Qi-Yu Zeng, Fan Zhang, Jian-Hui Zhang, Zhoufei Hei, Zi-Han Li, Meng-Han Huang, Pengfei Fang, En-Duo Wang, Xiao-Jian Sun, Xiao-Long Zhou

**Affiliations:** 1 State Key Laboratory of Molecular Biology, CAS Center for Excellence in Molecular Cell Science, Shanghai Institute of Biochemistry and Cell Biology, Chinese Academy of Sciences, University of Chinese Academy of Sciences, Shanghai, China; 2 Shanghai Institute of Hematology, State Key Laboratory of Medical Genomics, National Research Center for Translational Medicine, Ruijin Hospital Affiliated to Shanghai Jiao Tong University School of Medicine, Shanghai, China; 3 Key Laboratory of Systems Health Science of Zhejiang Province, School of Life Science, Hangzhou Institute for Advanced Study, University of Chinese Academy of Sciences, Hangzhou, China; 4 State Key Laboratory of Bioorganic and Natural Products Chemistry, Center for Excellence in Molecular Synthesis, Shanghai Institute of Organic Chemistry, Chinese Academy of Sciences, Shanghai, China; 5 School of Chemistry and Materials Science, Hangzhou Institute for Advanced Study, University of Chinese Academy of Sciences, Hangzhou, China

**Keywords:** aminoacyl-tRNA synthetase, aminoacylation, tRNA, translation

## Abstract

Aminoacyl-tRNA synthetases (aaRSs) are essential components for mRNA translation. Two sets of aaRSs are required for cytoplasmic and mitochondrial translation in vertebrates. Interestingly, *TARSL2* is a recently evolved duplicated gene of *TARS1* (encoding cytoplasmic threonyl-tRNA synthetase) and represents the only duplicated aaRS gene in vertebrates. Although TARSL2 retains the canonical aminoacylation and editing activities *in vitro*, whether it is a true tRNA synthetase for mRNA translation *in vivo* is unclear. In this study, we showed that *Tars1* is an essential gene since homozygous *Tars1* KO mice were lethal. In contrast, when *Tarsl2* was deleted in mice and zebrafish, neither the abundance nor the charging levels of tRNA^Thr^s were changed, indicating that cells relied on *Tars1* but not on *Tarsl2* for mRNA translation. Furthermore, *Tarsl2* deletion did not influence the integrity of the multiple tRNA synthetase complex, suggesting that Tarsl2 is a peripheral member of the multiple tRNA synthetase complex. Finally, we observed that *Tarsl2-*deleted mice exhibited severe developmental retardation, elevated metabolic capacity, and abnormal bone and muscle development after 3 weeks. Collectively, these data suggest that, despite its intrinsic activity, loss of *Tarsl2* has little influence on protein synthesis but does affect mouse development.

Aminoacyl-tRNA synthetases (aaRSs) are ubiquitously expressed housekeeping proteins that are critical for catalyzing the ligation of tRNAs with cognate amino acids, thus providing the aminoacyl-tRNA building blocks for ribosomal protein synthesis (1, 2). In addition to tRNA aminoacylation, approximately one-half of extant aaRSs have evolved with a proofreading (editing) function, enabling them to remove tRNAs that are improperly charged (such as Ser-tRNA^Thr^) (3, 4). Thus, aminoacylation and editing activities are two conserved canonical functions of aaRSs.

In vertebrates, due to presence of cytoplasmic and mitochondrial protein synthesis, there are two mRNA translation systems, requiring two sets of aaRSs, in either cytoplasm or mitochondria (5, 6, 7). In mammalian cells, two sets of aaRSs are usually encoded by two separate aaRS genes, except *KARS1* and *GARS1*, in both cases one gene encodes two different protein isoforms *via* alternative mRNA splicing (8, 9) or translational reinitiation (10). Accordingly, there are in total 37 aaRS genes in human cells (5). One typical feature of mammalian aaRSs is the existence of a multiple tRNA synthetase complex (MSC) comprising nine aaRS activities (RARS1, LARS1, IARS1, MARS1, EPRS1, QARS1, DARS1, and KARS1) and three nonenzymatic auxiliary factors (p43/AIMP1, p38/AIMP2, and p18/AIMP3) (11, 12, 13). The evolution of MSC formation is regarded as a progressive process with the appearance of simple entities in lower eukaryotes and complicated entities in higher eukaryotes. AaRSs from higher eukaryotes often have a N- or C-terminal extension than their bacterial or lower eukaryotic counterparts, including the glutathione S-transferase (GST) domain, the WHEP domain and other aaRS-specific ones (14). The primary sequences and topological structures of these extensions differ remarkably. It is generally found that these extensions are not involved in substrate binding and catalysis. However, they participate in protein–protein interaction, as in MSC assembly (14, 15). Interaction patterns between several components of MSC have been clarified by various methods including crosslinking, yeast two hybrids, or crystal structure determination (15, 16, 17). For example, the N-terminal extension of RARS1 forms two long conserved α-helixes each containing a leucine-zipper (LZ), which promotes its hydrophobic interaction with the N-terminal α-helix of p43. Meanwhile, the N-terminal extension of RARS1 is also involved in interaction with QARS1 (17). In addition, mammalian aaRSs frequently perform additional functions distinct from tRNA charging and editing. These noncanonical functions include mTOR signaling, mRNA translation regulation, inflammation, gene transcription, virus replication, among others (18, 19). In line with these observations, one of the rationales of MSC formation is to coordinately integrate both canonical and noncanonical functions of aaRSs. Once released from the MSC, aaRSs are ready to fulfill their regulatory roles (2, 18, 19).

Cytoplasmic and mitochondrial threonyl-tRNA synthetases (ThrRSs) in eukaryotic cells are encoded by *TARS1* and *TARS2*, respectively (5, 20). Interestingly, a *TARSL2* (*TARS3*) gene derived from the duplication of the *TARS1* gene and encoding a third ThrRS-like protein has been identified in vertebrates (20, 21, 22, 23, 24, 25). The most striking difference between TARS1 and TARSL2 is the distinct N-terminal extensions of the two proteins. The extension of TARSL2 is larger (161-aa in length) than that of TARS1 (82-aa in length), the former carrying sequences from the N-terminal extensions of RARS1 and TARS1 (22, 23). The N-terminal extension of TARSL2 readily facilitates TARSL2 incorporation into the MSC. We have previously demonstrated that the two LZs in the RARS1-homologous peptide of the N-terminal extension are critical for MSC incorporation. By performing both yeast two-hybrid and coimmunoprecipitation assays, we revealed that TARSL2 interacted with both RARS1 and p43 in the MSC (23). Notably, the N-terminal extension of RARS1 interacted with p43 in the MSC (17). Although the structure of *Escherichia coli* ThrRS was resolved more than 2 decades ago (26), the structure of full-length eukaryotic ThrRS has never been elucidated (27). To date, only half of the eukaryotic cytoplasmic and mitochondrial ThrRSs, including the aminoacylation domain and the tRNA-binding domain, have been characterized (27, 28). The three-dimensional structures of the N-terminal extensions of TARS1 and TARSL2 remain unknown. However, the catalytic domains of TARS1 and TARSL2, including the N1 (editing regulation) (29), N2 (editing), aminoacylation, and C-terminal tRNA-binding domains, are highly conserved (22). We have previously reported that the aminoacylation and editing sites are active in TARSL2 and that they charge tRNA^Thr^ and edit mischarged Ser-tRNA^Thr^ *in vitro* (22). However, one of the most fundamental questions about *TARSL2*, namely whether *TARSL2* has evolved to perform canonical functions *in vivo*, is unclear. In other words, due to presence of *TARSL2* in vertebrate, whether *TARS1* is an essential gene for normal development and growth is an open question.

In this work, we modeled the structures of TARS1 and TARSL2, and revealed that their N-terminal extensions adopt different architectures, while the structures of other domains are very similar. We also showed that *Tars1* is essential for embryonic development in mice and thus cannot be knocked out; in contrast, *Tarsl2* can be deleted. The absence of *Tarsl2* in mice had no effect on the abundance or aminoacylation level of tRNA^Thr^ or other noncognate tRNAs, but a disruption of growth was observed with alterations in metabolism, in bone and muscle development. Similarly, *Tarsl2* can also be deleted without exerting an obvious effect on tRNA^Thr^ aminoacylation, development, or angiogenesis in zebrafish. In summary, our data suggest that the recently emerged *Tarsl2* is not involved in tRNA aminoacylation under physiological conditions and therefore cannot replace the essential function of *Tars1*. The precise function of *Tarsl2* awaits further investigation.

## Results

### TARSL2 and TARS1 have a similar structure, except for their N-terminal extensions

**Figure 1:**
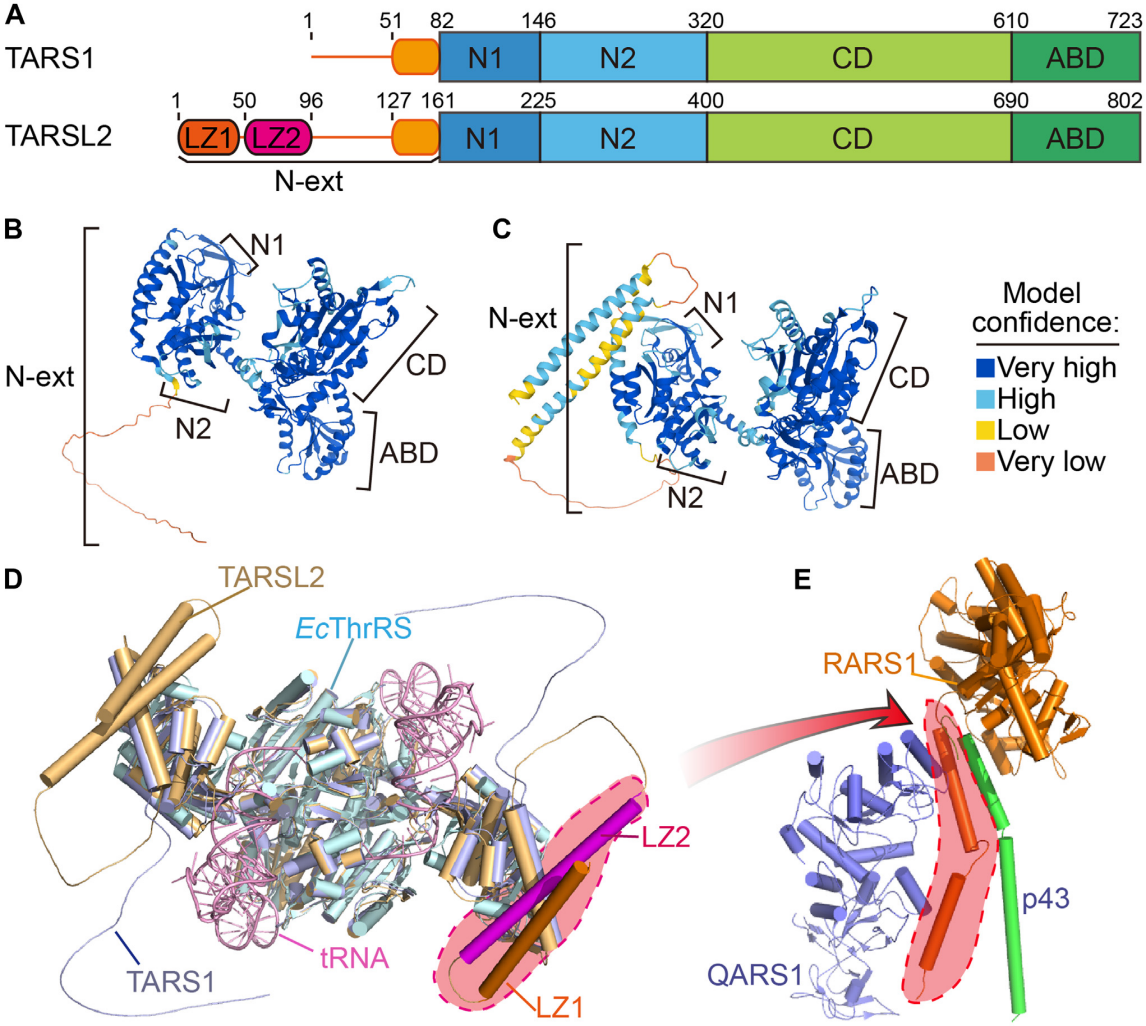
**The topology of TARSL2 in the N-terminal extension differs from that of TARS1.***A*, the domains of TARS1 and TARSL2. *B*, the TARS1 structure model was predicted by AlphaFold 2. *C*, the TARSL2 structure model was predicted by AlphaFold 2. In (*B* and *C*), the structures are colored on the basis of the confidence of each model prediction. *D*, the structural models of TARS1 and TARS2 are superimposed onto the *E. coli* ThrRS-tRNA structure (PDB ID 1QF6). TARS1 is *light blue*. TARSL2 is *wheat*; however, the leucine zippers in one chain are highlighted in *orange* and *magenta*. *E. coli* ThrRS is *cyan*, and tRNA is *pink*. *E*, the leucine zippers of RARS1 are highlighted with a *red dashed line* in the RARS1–QARS1–p43 complex structure (PDB ID 4R3Z). ABD, anticodon binding domain; CD, catalytic domain; LZ, leucine zipper; N-ext, N-terminal extension; ThrRS, threonyl-tRNA synthetase.

*TARSL2* results from the duplication of the *TARS1* gene during evolution. TARSL2 and TARS1 have very similar domains/regions, including an N-terminal extension and two N1 and N2 domains related to editing activity, an aminoacylation catalytic domain, and an anticodon binding domain (Fig. 1*A*). However, the TARSL2 N-terminal extension differs from that of TARS1 in that the former is twice as long, the additional region carrying two LZs (Fig. 1*A*). Due to the significant level of accuracy achieved by a deep learning-based approach to predict the 3D structure of proteins (30), we were able to analyze the structures of TARS1 and TARSL2 predicted by AlphaFold 2 even without the full-length crystal structures (31). Except for the disordered regions (residues 1-51 of TARS1 and residues 96-127 of TARSL2), which showed low model confidence, the models of most structures, including the helix in the C-end of the extension region and the other four domains, were predicted with a high degree of confidence and the two LZs of TARSL2 were predicted with a medium degree of confidence (Fig. 1, *B*–*D*). The TARS1 and TARSL2 models could be superimposed on the crystal structure of the *E. coli* ThrRS–tRNA complex (Fig. 1*D*), consistent with our previous finding that TARSL2 exhibits intrinsic tRNA aminoacylation and editing activities (22). The two LZs of TARSL2, which are absent in TARS1, were predicted to form a hairpin structure and be attached to the editing domain (Fig. 1, *C* and *D*). We have previously shown that the two LZs were very similar to the N terminus of RARS1, which interacts with p43 and QARS1 (Fig. 1*E*), and that TARSL2 was incorporated into the MSC through these two LZs (23). When the two LZs from the TARSL2 N-terminal extension region are assembled into the MSC, conformational changes are likely to occur, allowing TARSL2 interactions with p43, QARS1, or RARS1 (Fig. 1*E*). These findings indicate that TARSL2 carries an additional extension and that this extension binds the MSC, which is the only structural difference between TARSL2 and TARS1.

### *Tars1* is an essential gene and cannot be replaced by *Tarl2in vivo*

**Figure 2:**
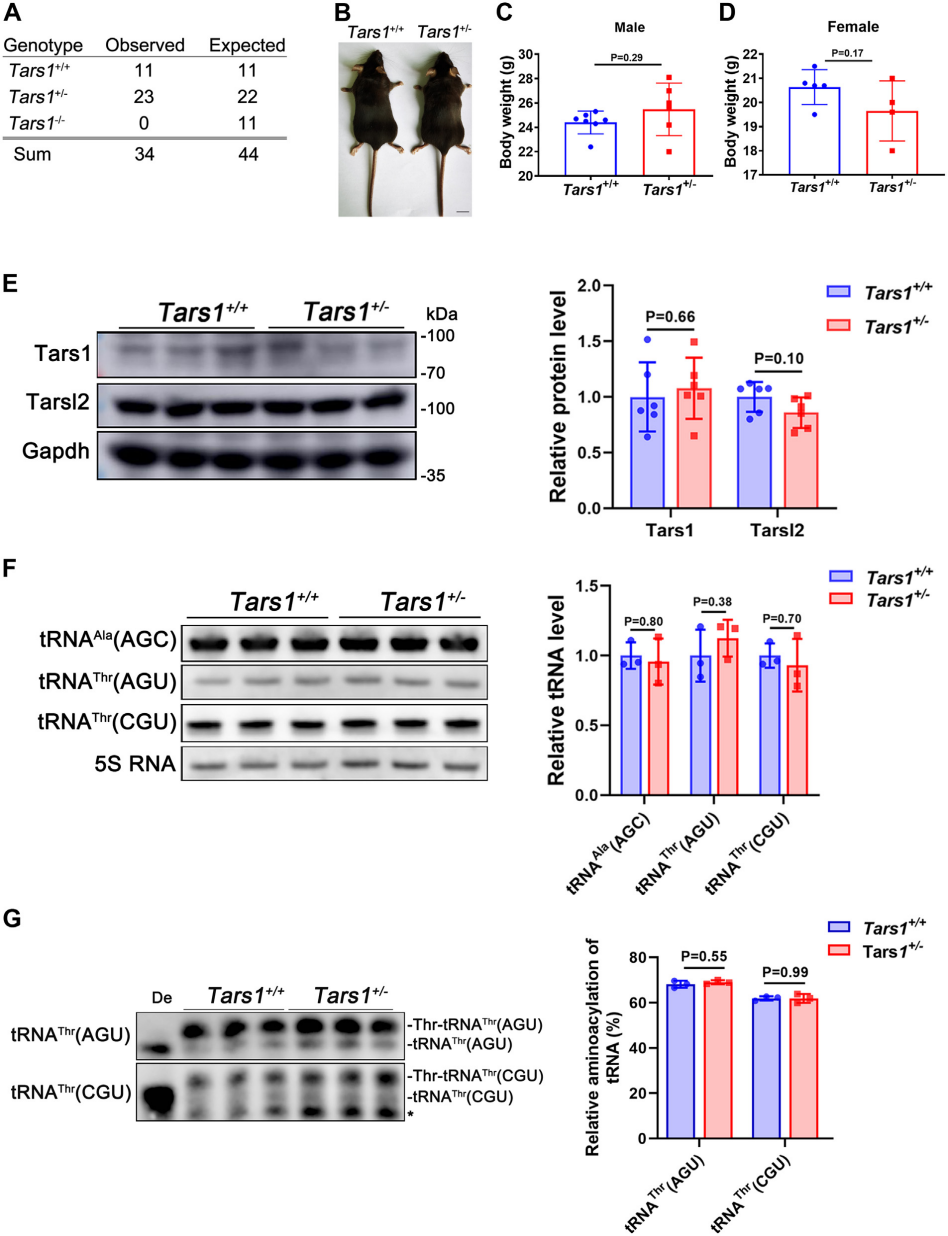
***Tars1* is indispensable *in vivo*.***A*, statistical analysis showing the genetic features of the *Tars1*-KO mice (n = 34). *B*, body features of the 10-week-old *Tars1*^+/+^ and *Tars1*^+/−^ mice. The scale bar represents 1 cm. *C*, statistical analysis showing the weights of the 16-week-old *Tars1*^+/+^ (n = 7) and *Tars1*^+/−^ (n = 6) male mice. *D*, statistical analysis showing the weights of the 16-week-old *Tars1*^+/+^ (n = 5) and *Tars1*^+/−^ (n = 4) female mice. *E*, protein abundance of the 8-week-old Tars1 and Tarsl2 in *Tars1*^+/+^ and *Tars1*^+/−^ mouse muscle as detected by Western blot. Gapdh was used as the loading control. *F*, levels of tRNA^Ala^(AGC), tRNA^Thr^(AGU), and tRNA^Thr^(CGU) in muscle tissues of the 8-week-old *Tars1*^+/+^ and *Tars1*^+/−^ mice. 5S RNA was as the loading control. *G*, aminoacylation levels of tRNA^Thr^(AGU) and tRNA^Thr^(CGU) in muscle tissues of the 8-week-old *Tars1*^+/+^ and *Tars1*^+/−^ mice. ∗, a nonspecific band. De: tRNA deaminoacylation control. Error bars in (*C*–*G*) represent the mean ± SD with the *p* values indicated (two-tailed Student’s *t* test).

To understand whether *Tars1* is an essential gene or can be substituted with *Tarsl2 in vivo*, we initially tried to knock out the *Tars1* gene in the NIH3T3 cell line using the CRISPR/Cas9 method. However, despite extensive efforts, we were unable to obtain a null allele, implying that *Tars1* may be essential for cell viability. To directly explore a potential pivotal role for *Tars1 in vivo*, we purchased commercially available heterozygous *Tars1* gene-KO mice (C57BL/6N-*Tars*^em1cyagen^) from Cyagen. Crossing the mice revealed that only WT (*Tars1*^+/+^) and heterozygous (*Tars1*^+/−^) mice, but not homozygous (*Tars1*^−/−^) *Tars1*-deletion mice, were viable and they were obtained at a 1:2 proportion (Fig. 2*A*). The growth and body weight of *Tars1*^+/+^ and *Tars1*^+/−^ mice were comparable (Fig. 2, *B*–*D*). No significant difference in the protein level of Tars1 was observed in muscle tissue between *Tars1*^+/+^ and *Tars1*^+/−^ mice (Fig. 2*E*). Similarly, the steady-state protein abundance of Tarsl2 in *Tars1*^+/+^ and *Tars1*^+/−^ mice did not differ (Fig. 2*E*), suggesting that *Tarsl2* gene expression was not stimulated. The steady-state amounts of tRNA^Thr^(AGU) and tRNA^Thr^(CGU) and their charging levels in muscle tissue were comparable between *Tars1*^+/+^ and *Tars1*^+/−^ mice (Fig. 2, *F* and *G*). All these data showed that *Tars1* is an essential gene for cell viability and mouse survival *in vivo* and deletion of one copy of *Tars1* did not lead to haploinsufficiency. Furthermore, the results suggest that *Tars1* cannot be replaced by *Tarsl2 in vivo*.

### Tarsl2 is not critical for canonical tRNA aminoacylation *in vivo*

**Figure 3:**
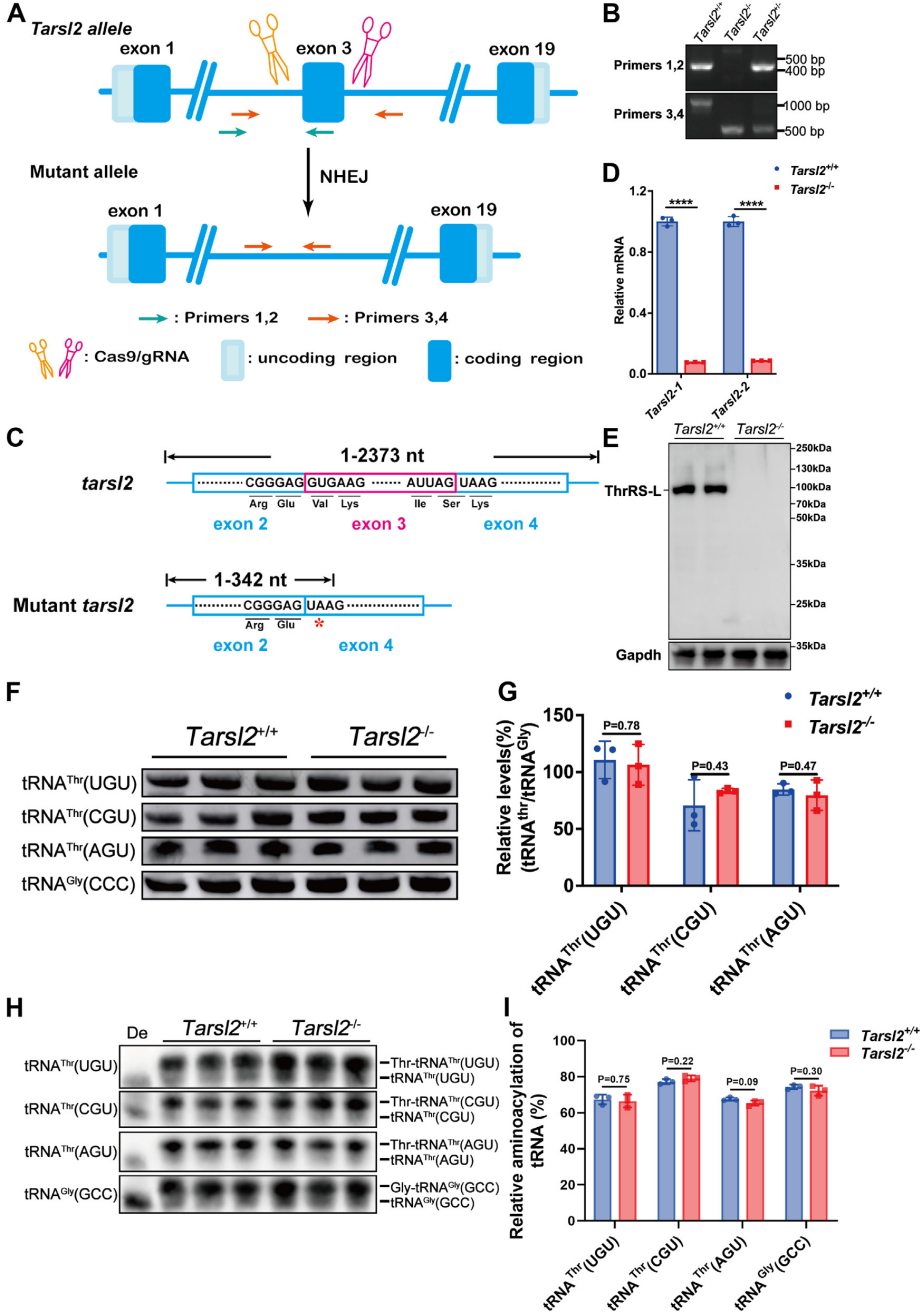
**Loss of *Tarsl2* exerts no effect on the steady-state or charging of tRNA**^**Thr**^**s *in vivo.****A*, schematic showing *Tarsl2*-mutant mouse construction by CRISPR/Cas9 gene editing technology. Two sgRNAs were designed around exon 3 to delete a sequence fragment. Primers 1 and 2 and Primers 3 and 4 were used to detect DNA mutations. *B*, PCR detection of the *Tarsl2*^+/+^, *Tarsl2*^+/−^, and *Tarsl2*^−/−^ mouse genotypes using Primers 1 and 2 and Primers 3 and 4. *C*, sequencing results of *Tarsl2*^+/+^ and *Tarsl2*^−/−^ mice. In the mutant mice, *Tarsl2* mRNA translation was prematurely terminated at nt 342. *D*, *Tarsl2* mRNA levels in the muscle of the 6-week-old *Tarsl2*^+/+^ and *Tarsl2*^−/−^ mice, as determined by RT‒PCR with two pairs of primers. *E*, protein abundance of Tarsl2 in the 6-week-old *Tarsl2*^+/+^ and *Tarsl2*^−/−^ mouse muscle, as detected by Western blotting. Gapdh was used as the loading control. *F* and *G*, levels of tRNA^Thr^(UGU), tRNA^Thr^(CGU), and tRNA^Thr^(AGU) in the muscle tissues of the 6-week-old *Tarsl2*^+/+^ and *Tarsl2*^−/−^ mice. tRNA^Gly^(CCC) was included as the loading control. *H* and *I* Aminoacylation levels of tRNA^Thr^(UGU), tRNA^Thr^(CGU), and tRNA^Thr^(AGU) in the muscle tissues of the 6-week-old *Tarsl2*^+/+^ and *Tarsl2*^−/−^ mice. tRNA^Gly^(CCC) was included as the control. De: tRNA deaminoacylation control. ∗∗∗∗*p* < 0.0001. Error bars in (*G* and *I*) represent the mean ± SD with the *p* values indicated (two-tailed Student’s *t* test).

To explore the function of *Tarsl2 in vivo*, we successfully constructed homozygous *Tarsl2* gene-KO (*Tarsl2*^−/−^) mice using CRISPR/Cas9-mediated gene editing. The survival of the *Tarsl2*^−/−^ mice suggests that *Tarsl2* is not essential *in vivo*, consistent with our previous findings obtained by constructing the *Tarsl2*-deleted NIH3T3 cell line (23). In *Tarsl2*^−/−^ mice, a genomic fragment including exon 3 of *Tarsl2* was deleted (Fig. 3*A*), leading to a truncated allele, which was confirmed by two sets of primers (Fig. 3*B*). Premature termination of translation of the mRNA transcribed from this allele occurrs at position 342, encoding a truncated protein of 113 amino acids (Fig. 3*C*). RT‒quantitative PCR (qPCR) analyses using two different primer sets showed that *Tarsl2* mRNA was significantly downregulated compared to WT mice, suggesting its degradation by nonsense-mediated mRNA decay (Fig. 3*D*). In addition, Tarsl2 protein was absent in *Tarsl2*^−/−^ mice (Fig. 3*E*).

*Tarsl2* is most highly expressed in mouse muscle and heart tissue (22). We have previously proposed that, given its nuclear localization, Tarsl2 may be involved in quality control of tRNA^Thr^ in the nucleus and thus contribute to the amount of cytoplasmic tRNA^Thr^ (22, 23). However, a Northern blot analysis showed that, in muscle, the steady-state levels of three cytoplasmic tRNA^Thr^ isoacceptors [tRNA^Thr^(UGU), tRNA^Thr^(CGU), and tRNA^Thr^(AGU)] was not changed after *Tarsl2* depletion (Fig. 3, *F* and *G*). Furthermore, the aminoacylation levels for tRNA^Thr^(UGU), tRNA^Thr^(CGU), and tRNA^Thr^(AGU) in muscle tissues from WT and *Tarsl2*^*−/−*^ mice were examined and compared in acidic polyacrylamide/urea gel system. The results showed little difference in the aminoacylation levels of the three tRNA^Thr^s between the two mice (Fig. 3, *H* and *I*). These results strongly coincided with our previous hypothesis (23) suggesting that despite its ability to catalyze canonical aminoacylation *in vitro*, Tarsl2 is not critical for mRNA translation *in vivo*.

### Tarsl2 is likely a peripheral member of the MSC

**Figure 4:**
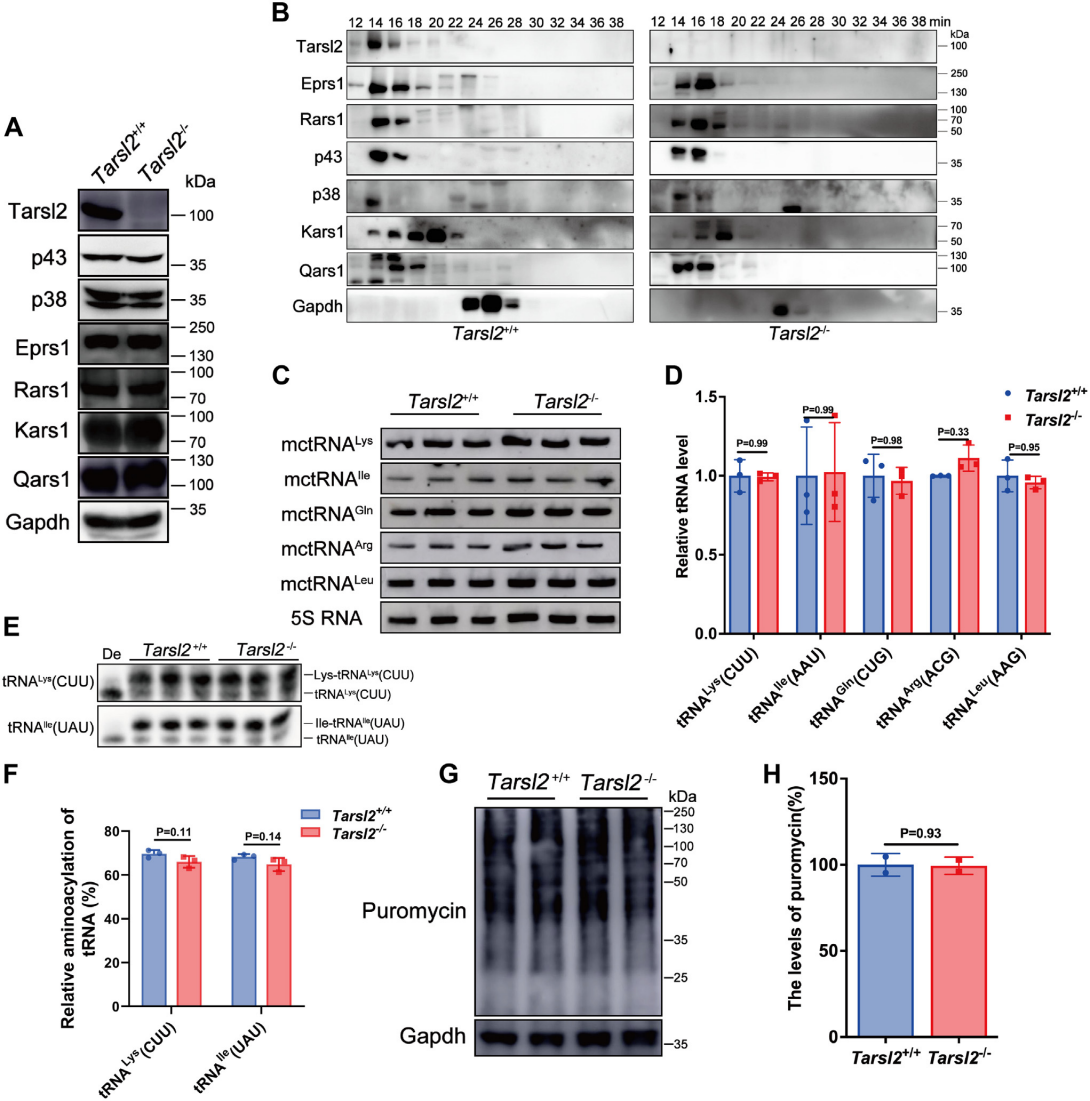
**Deletion of *Tarsl2* exerts no effect on MSC integrity or tRNA aminoacylation *in vivo*.***A*, protein levels of various MSC components in the 6-week-old *Tarsl2*^+/+^ and *Tarsl2*^−/−^ mouse muscle. *B*, incorporation of several MSC components in the 6-week-old *Tarsl2*^+/+^ and *Tarsl2*^−/−^ mouse muscle lysates. *C* and *D*, levels of tRNAs [tRNA^Lys^(CUU), tRNA^Ile^(AAU), tRNA^Gln^(CUG), tRNA^Arg^(ACG), and tRNA^Leu^(AAG)] in the 6-week-old *Tarsl2*^+/+^ and *Tarsl2*^−/−^ mouse muscle. *E* and *F*, aminoacylation levels of tRNA^Lys^(CUU) and tRNA^Ile^(UAU) in the 6-week-old *Tarsl2*^+/+^ and *Tarsl2*^−/−^ mouse muscle. De: tRNA deaminoacylation control. *G* and *H*, global protein synthesis rate of the 6-week-old *Tarsl2*^+/+^ and *Tarsl2*^−/−^ mouse MEFs, as determined by puromycin incorporation assay. Gapdh was included as the loading control. Error bars in (*D*, *F*, and *H*) represent the mean ± SD with the *p* values indicated (two-tailed Student’s *t* test). MEF, mouse embryonic fibroblast; MSC, multiple tRNA synthetase complex.

We have already reported that TARSL2 is a *bone fide* component of the MSC by interacting with RARS1 and p43 (23). Thus, we measured the levels of both proteins and assessed their incorporation into the MSC in *Tarsl2*^*−/−*^ mice. We found little difference in the steady state protein levels of several mouse cytoplasmic aaRSs (including Eprs1, Rars1, Kars1, Qars1, and Tars1) or that of two cofactors (p38 and p43) between WT and *Tarsl2*^*−/−*^ mice (Fig. 4*A*). We then sought to identify a role for Tarsl2 in maintaining the integrity of the MSC, notably through the incorporation of Rars1 and p43. For this purpose, muscle tissue lysate was fractionated by gel filtration chromatography. The data showed that after depletion of Tarsl2, several MSC components, including Rars1, p43, Qars1, and Eprs1, were copurified with the fractions containing the MSC, suggesting that Tarsl2 does not play a direct role in MSC assembly or in the incorporation of Rars1 and p43 in the MSC (Fig. 4*B*). These data suggested that, despite a direct interaction between Tarsl2 and p43 or between Tarsl2 and Rars1, the absence of Tarsl2 does not influence the incorporation of Rars1 or p43 into the MSC, which is consistent with the accepted role of p38 as the core scaffolding protein of the MSC (32) and the fact that Rars1 or p43 interacts with multiple proteins in the MSC (17). We further studied the steady-state abundance of several cytoplasmic tRNAs, including tRNA^Lys^(CUU), tRNA^Ile^(AAU), tRNA^Gln^(CUG), tRNA^Arg^(ACG), and tRNA^Leu^(AAG), which are aminoacylated by various MSC components. No significant difference was observed between WT and *Tarsl2*^*−/−*^ mice (Fig. 4, *C* and *D*). Furthermore, the aminoacylation levels of tRNA^Lys^(CUU) and tRNA^Ile^(UAU) were unchanged (Fig. 4, *E* and *F*). The global protein synthesis rate, as measured by puromycin incorporation assay, was comparable between WT and *Tarsl2*^*−/−*^ mice (Fig. 4, *G* and *H*), suggesting that the deletion of *Tarsl2* has little effect on the rate of translation *in vivo*. Altogether, these data suggest that Tarsl2 does not impact on the integrity of MSC *in vivo* and is likely to be a peripheral but not a core member of the MSC.

### Tarsl2 knockout affects the growth and metabolism of mice

**Figure 5:**
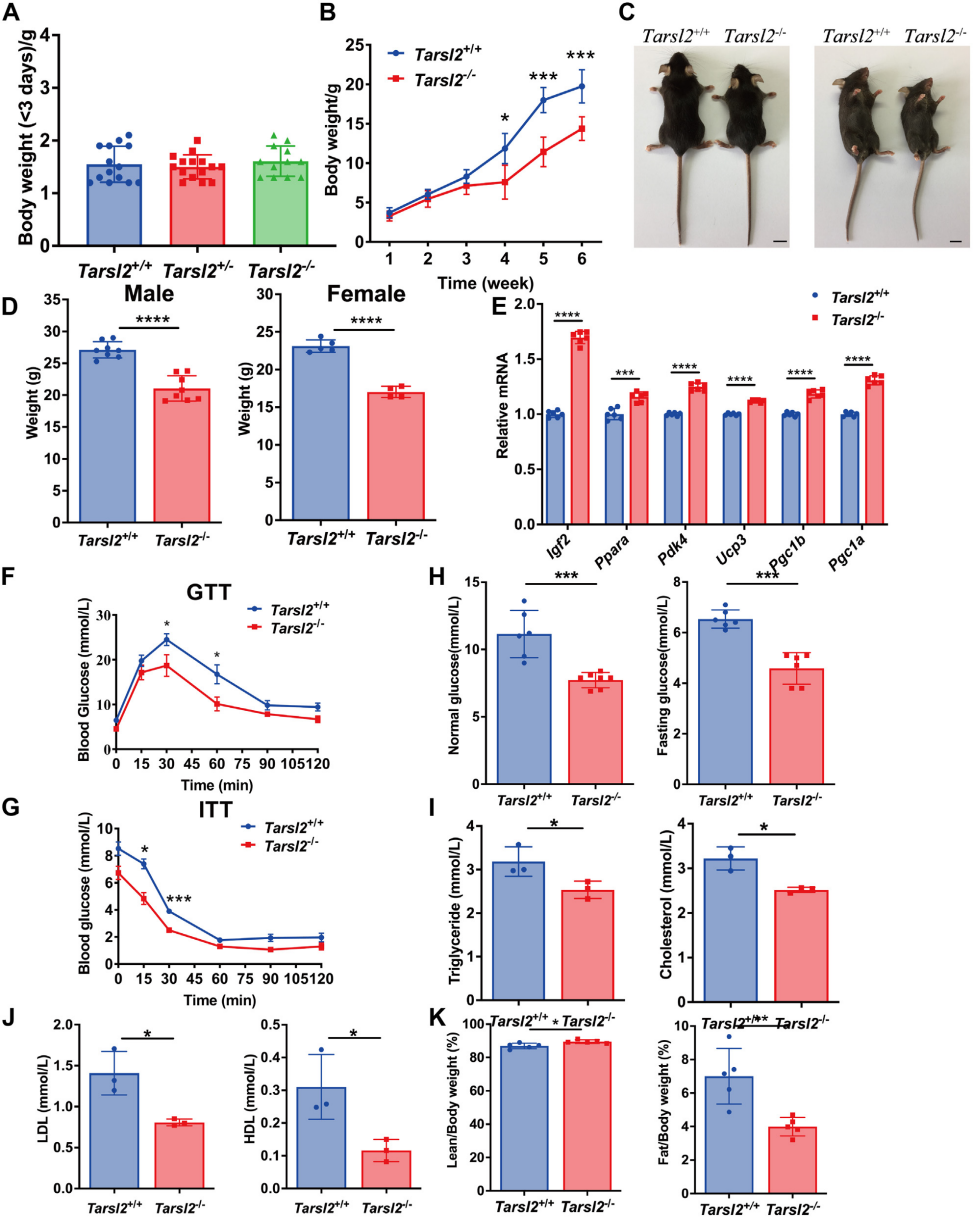
**Deletion of *Tarsl2* affects mouse growth and metabolism**. *A*, weight of *Tarsl2*^+/+^ (n = 14), *Tarsl2*^+/−^ (n = 15), and *Tarsl2*^−/−^ (n = 12) mice within 3 days of birth. *B*, growth curves of *Tarsl2*^+/+^ (n = 13) and *Tarsl2*^−/−^ mice (n = 3). *C*, body features of 10-week-old *Tarsl2*^+/+^ and *Tarsl2*^−/−^ mice. The scale bar represents 1 cm. *D*, statistical analysis showing the weights of the 16-week-old *Tarsl2*^+/+^ (n = 8) and *Tarsl2*^−/−^ (n = 8) male (*left*) and *Tarsl2*^+/+^ (n = 5) and *Tarsl2*^−/−^ (n = 4) female (*right*) mice. *E*, relative RNA levels of several metabolism-related genes in the 6-week-old *Tarsl2*^+/+^ and *Tarsl2*^−/−^ mouse muscle, as detected by RT‒PCR. *F*, blood glucose levels in the 6-week-old *Tarsl2*^+/+^ (n = 8) and *Tarsl2*^−/−^ (n = 5) mice after 16 h of starvation, as determined by GTT. *G*, blood glucose levels of the 6-week-old *Tarsl2*^+/+^ (n = 3) and *Tarsl2*^−/−^ (n = 3) mice after 5 h of starvation, as detected by ITT. *H*, blood glucose levels of the 6-week-old *Tarsl2*^+/+^ (n = 6) and *Tarsl2*^−/−^ (n = 7) mice were detected after satiety (*left*) and after 16 h of starvation (*right*). *I*, levels of triglyceride (*left*) and cholesterol (*right*) in the blood of the 6-week-old *Tarsl2*^+/+^ (n = 3) and *Tarsl2*^−/−^ (n = 3) mice. *J*, levels of LDL (*left*) and HDL (*right*) in the blood of the 6-week-old *Tarsl2*^+/+^ (n = 3) and *Tarsl2*^−/−^ (n = 3) mice. *K*, body fat percentage of the 6-week-old *Tarsl2*^+/+^ (n = 5) and *Tarsl2*^−/−^ (n = 5) mice, measured on the basis of fat/body weight (*left*) and lean body weight (*right*). ∗∗∗∗*p* < 0.0001, ∗∗∗*p* < 0.001, ∗∗*p* < 0.01 and ∗*p* < 0.05. The error bar represents the mean ± SD with the *p* values indicated (two-tailed Student’s *t* test).

*Tarsl2*^+/+^, *Tarsl2*^+/−^, and *Tarsl2*^−/−^ mice displayed comparable body weights at birth (Fig. 5*A*), suggesting that Tarsl2 is not involved in embryonic development. However, weight and size differences between *Tarsl2*^+/+^ and *Tarsl2*^−/−^ mice gradually increased, becoming obvious (Fig. 5, *B* and *C*) after 3 weeks and maintained throughout the mouse lifespan (Fig. 5*D*). These results suggested that the lack of Tarsl2 leads to developmental defects in mice.

Our previous study showed that *Tarsl2* was most highly expressed in mouse muscle; therefore, we performed an RNA-seq analysis with *Tarsl2*^+/+^ and *Tarsl2*^−/−^ mouse muscle tissue. The results showed that the expression of metabolism-related genes in the muscle was higher in the *Tarsl2*^−/−^ mice than in the *Tarsl2*^+/+^ mice (Fig. S1). Indeed, a RT‒qPCR analysis confirmed the upregulation of several well-established metabolism-related genes, including *Igf2*, *Ppara*, *Pdk4*, *Ucp3*, *Pgc1a*, and *Pgc1b* (33, 34, 35) (Fig. 5*E*). These results suggested that Tarsl2 may be involved in metabolism. Therefore, the effects of Tarsl2 on glucose homeostasis and insulin sensitivity were further determined by intraperitoneal glucose and insulin tolerance tests. We found that a lack of *Tarsl2* led to glucose and insulin tolerance (Fig. 5, *F* and *G*). Normal and fasting plasma glucose levels were measured and the results showed that their levels were obviously lower in the *Tarsl2*^−/−^ mice than in the *Tarsl2*^+/+^ mice (Fig. 5*H*). Subsequently, the triglyceride, cholesterol, LDL, and HDL were determined after isolating the plasma blood and all of these compounds were lower in the *Tarsl2*^−/−^ mice than in the *Tarsl2*^+/+^ mice (Fig. 5, *I* and *J*). Moreover, a body fat analysis showed that the *Tarsl2*^−/−^ mice were leaner than the *Tarsl2*^+/+^ mice (Fig. 5*K*). Collectively, these results indicated a functional requirement for Tarsl2 in mouse growth and metabolism.

### *Tarsl2* deletion causes abnormal development of bone and muscle

**Figure 6:**
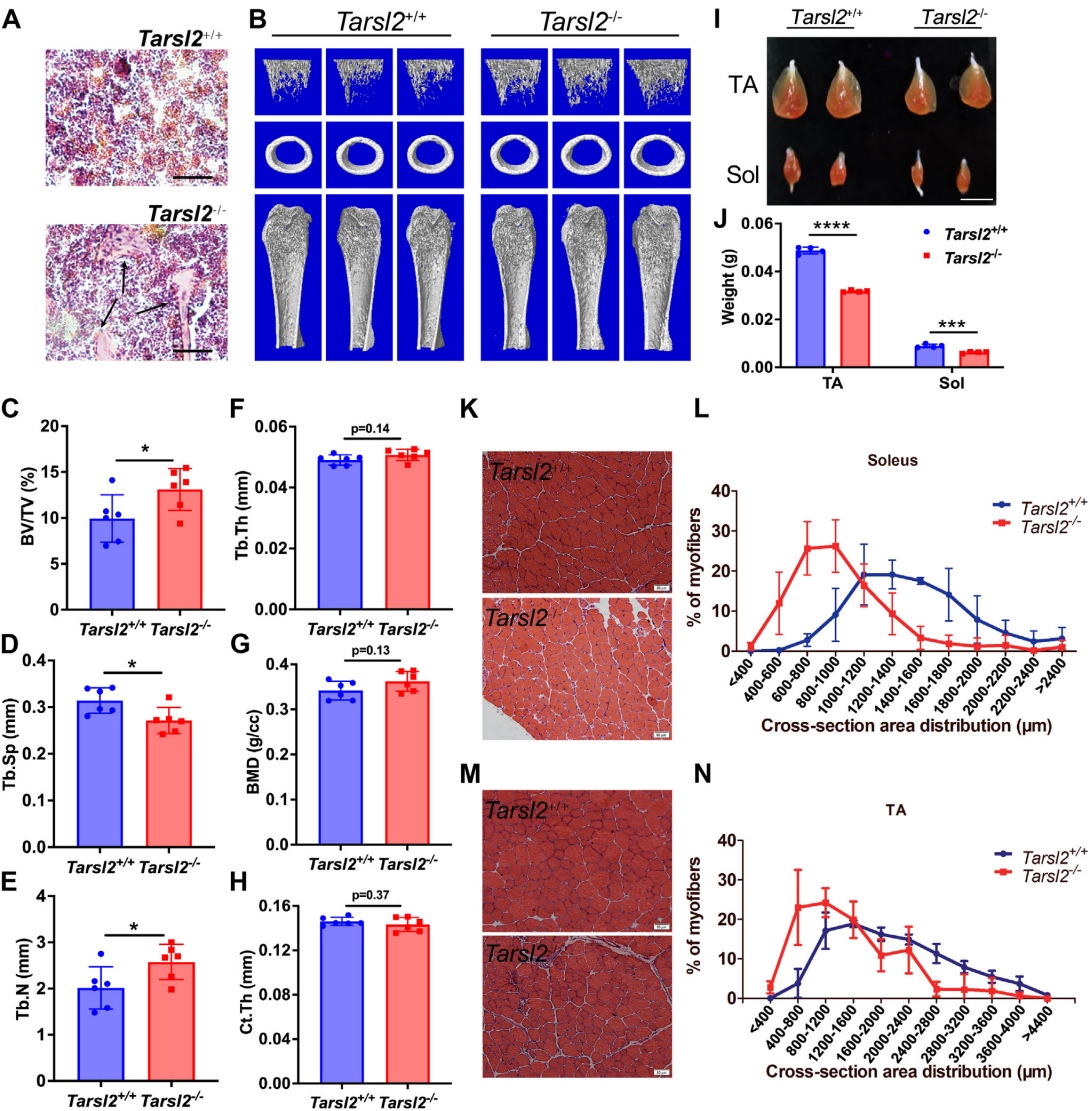
***Tarsl2* deletion affects the development of bone and muscle.***A*, H&E staining of bone sections from 8-week-old *Tarsl2*^+/+^ and *Tarsl2*^−/−^ mice. The *black arrow* indicates the abnormal trabeculae in the tibia of the *Tarsl2*^−/−^ mice. The scale bar represents 40 mm. *B*, three-dimensional μ-CT images of the trabecular bone in distal femurs isolated from 8-week-old male *Tarsl2*^+/+^ and *Tarsl2*^−/−^ mice (n = 6). *C*–*H*, the μ-CT results in (*B*) were quantitatively analyzed to determine bone volume fraction (BV/TV), trabecular thickness (Tb.Th), the number of trabeculae (TB.N), the separation of trabeculae (Tb.Sp), bone mineral density (BMD), and cortical bone thickness (Ct.Th) (n = 6). *I*, comparisons of the tibialis anterior muscle and soleus muscle between the 6-week-old *Tarsl2*^+/+^ and *Tarsl2*^−/−^ mice. The scale bar represents 1 cm. *J*, the weight of the TA and soleus muscle of the *Tarsl2*^+/+^ and *Tarsl2*^−/−^ mice in (*I*) was measured. *K*, H&E staining of soleus cross sections from 6-week-old *Tarsl2*^+/+^ and *Tarsl2*^−/−^ mice. *L*, area of the soleus cross section from *Tarsl2*^+/+^ and *Tarsl2*^−/−^ mice. *M*, H&E staining of TA cross sections from 6-week-old *Tarsl2*^+/+^ and *Tarsl2*^−/−^ mice. *N*, area of TA cross sections of *Tarsl2*^+/+^ and *Tarsl2*^−/−^ mice. ∗∗∗∗*p* < 0.0001, ∗∗∗*p* < 0.001, and ∗*p* < 0.05. The error bar represents the mean ± SD with the *p* values indicated (two-tailed Student’s *t* test). TA, tibialis anterior.

*Tarsl2* deficiency leads to a decrease in mice size during development (Fig. 5*C*). Therefore, we assessed bone development in 8-week-old*Tarsl2*^+/+^ and *Tarsl2*^−/−^ mice. H&E staining showed ossified and clear bone trabeculae in the distal femur in the *Tarsl2*^−/−^ mice but not in the *Tarsl2*^+/+^ mice (Fig. 6*A*). This meant that bone development was abnormal in the *Tarsl2*-deficient mice. To determine the function of Tarsl2 in the skeletal system, a microquantitative computed tomography analysis was performed to compare changes in bone-related elements in the long bones of the *Tarsl2*^−/−^ mice and *Tarsl2*^+/+^ mice (Fig. 6*B*). Eight-week-old *Tarsl2*^−/−^ mice showed increased bone mass per tissue volume (BV/TV) compared to the *Tarsl2*^+/+^ mice (Fig. 6*C*). Further analysis revealed a difference in the trabecular separation of cancellous bone (Tb.Sp) and trabecular number (Tb.N) (Fig. 6, *D* and *E*), but there was no significant difference in trabecular thickness (Tb.Th), bone mineral density, or cortical bone thickness (Ct.Th) (Fig. 6, *F*–*H*). These results show that Tarsl2 deletion influences bone development.

Based on the high expression of *Tarsl2* in muscle, which is closely connected to metabolism, we further measured muscle development in *Tarsl2*^−/−^ mice and compared the results to those obtained with WT *Tarsl2*^+/+^ mice. Notably, the sizes of the tibialis anterior (TA) and soleus muscle (Sol) were significantly smaller in the *Tarsl2*^−/−^ mice than in the *Tarsl2*^+/+^ mice (Fig. 6, *I* and *J*). Subsequently, we examined whether the smaller muscle in *Tarsl2*-null mice was due to fiber size differences. Quantification of a cross-sectional myofiber area showed that the fiber size in both the TA and Sol was significantly smaller in the *Tarsl2*^−/−^ mice than in the *Tarsl2*^+/+^ littermates (Fig. 6, *K*–*N*). Thus, Tarsl2 plays a crucial role in muscle development.

### The tRNA^Thr^ aminoacylation level remains unaltered in *Tarsl2*-deleted zebrafish

**Figure 7:**
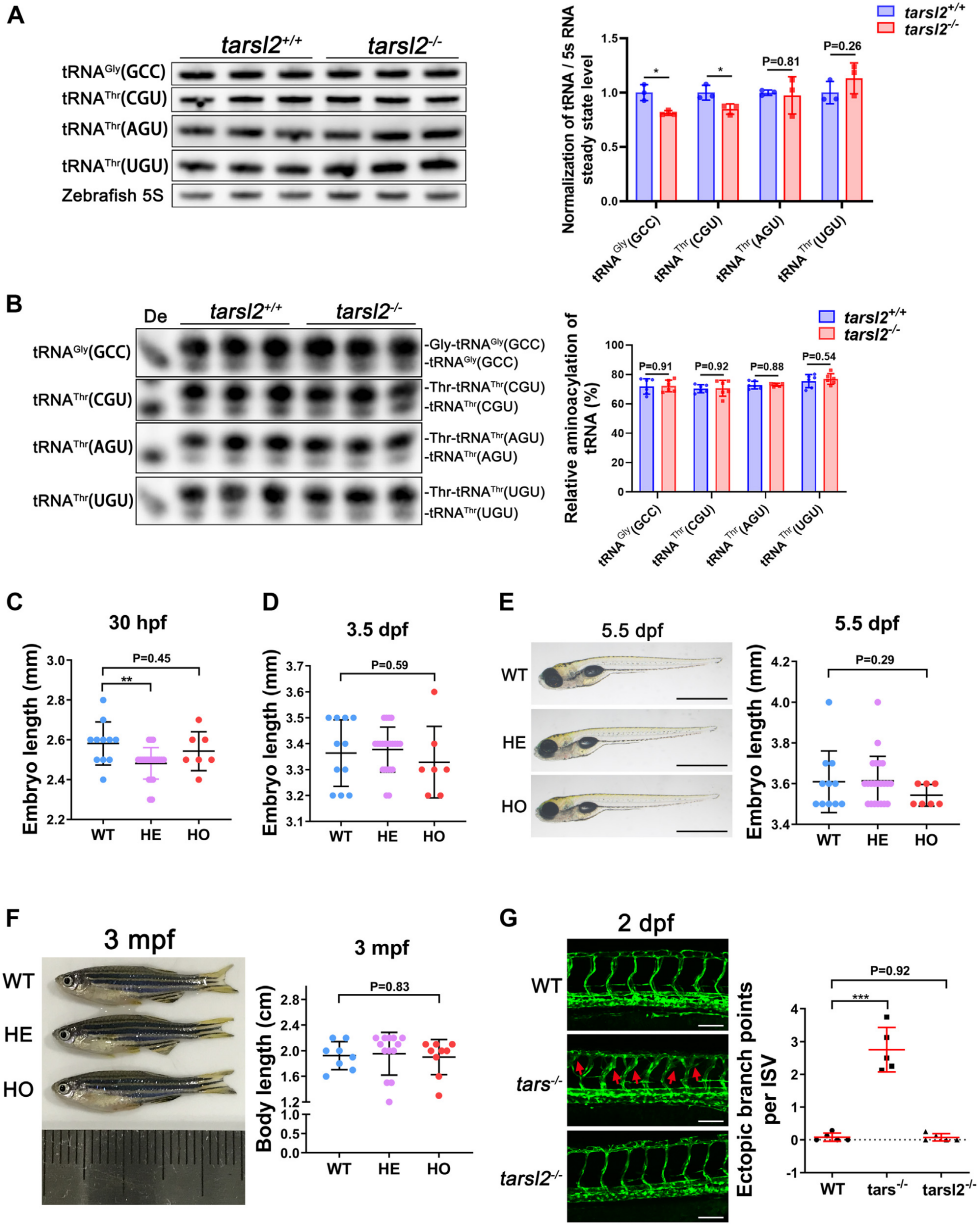
**The *tarsl2*-null zebrafish display a normal phenotype.***A*, levels of tRNA ^Gly^(GCC), tRNA^Thr^(CGU), tRNA^Thr^(AGU), and tRNA^Thr^(UGU) in WT and *tarsl2*^−/−^ zebrafish embryos, with quantification shown on the *right*. Zebrafish 5S RNA was included as the loading control. *B*, aminoacylation levels of tRNA^Gly^(GCC), tRNA^Thr^(CGU), tRNA^Thr^(AGU), and tRNA^Thr^(UGU) in WT and *tarsl2*^−/−^ zebrafish embryos, with the quantification analysis results shown on the right. De: tRNA deaminoacylation control. *C* and *D*, quantification analysis showing the body length of the WT, *tarsl2*^+/−^ and *tarsl2*^−/−^ embryos 30 hpf (*C*) and 3 dpf (*D*). *E*, images showing the body length of the WT, *tarsl2*^+/−^ and *tarsl2*^−/−^ embryos 5.5 dpf on the *left*, with quantification analysis shown on the right. The scale bar reprents 1 mm. *F*, representative images (*left*) and quantification (*right*) results showing the body length of the WT, *tarsl2*^+/−^ and *tarsl2*^−/−^ zebrafish 3 mpf. *G*, representative confocal microscopy images (*left*) and quantification analysis (*right*) of the ectopic branch points per ISV of the WT, *tars*^−/−^ and *tarsl2*^−/−^ embryos two dpf. The scale bar represents 100 μm. ∗∗*p* < 0.01 and ∗∗∗*p* < 0.001. The error bar represents the mean ± SD with the *p* values indicated (two-tailed Student’s *t* test).

Zebrafish Tarsl2 displays high sequence similarity (73.1%) with mouse Tarsl2. To understand whether *Tarsl2* has evolved with tRNA charging capacity and to explore the role of *tarsl2* in lower vertebrates, we used the CRISPR‒Cas9 system to establish a *tarsl2*-null (*tarsl2*^−/−^) zebrafish line that carries a 1 bp deletion (c.1356delG), which causes a frameshift after leucine codon 475 (L475) (Fig. S2*A*). This mutant Tarsl2 protein (p.L475fs∗23) was nonfunctional, as the catalytic domain (core) and the tRNA-binding domain were missing due to the premature stop codon. The RT‒qPCR results showed that the mRNA expression of *tarsl2* was markedly reduced in *tarsl2*^−/−^ embryos 5 days postfertilization (Fig. S2*B*), suggesting that the mutated mRNA was degraded by the nonsense-mediated mRNA decay pathway. We first compared the steady-state abundance of tRNA^Thr^ between WT and *tarsl2*^−/−^ fish. The amount of tRNA^Thr^(AGU) was significant but somewhat lower in the *tarsl2*^−/−^ fish, similarly to noncognate tRNA^Gly^(GCC); however, comparable tRNA^Thr^(AGU) and tRNA^Thr^(UGU) levels were observed (Fig. 7*A*). Moreover, tRNA^Thr^ charging between the two fish lines was similar, as determined by acidic Northern blot (Fig. 7*B*). The *tarsl2*^−/−^ zebrafish were viable and showed normal fertility. The body length of the *tarsl2*^−/−^ zebrafish was also similar to that of WT and heterozygous zebrafish in the early stage of embryonic development (Fig. 7, *C*–*E*) and in adults (Fig. 7*F*). Given that knocking out *tars*, the paralog of *tarsl2*, led to the acquisition of a stress-induced angiogenic phenotype (36), we next questioned whether this phenotype was acquired in our *tarsl2*^−/−^ embryos. We crossed the *tarsl2*^−/−^ mutant with a transgenic (Tg) line (flk1:EGFP) to visualize the development of blood vessels *in vivo*. As expected, *tars* KO but not *tarsl2* KO induced the acquisition of an ectopic angiogenic phenotype compared to WT embryos (Fig. 7, *G*). Taken together, these data demonstrate that, similar to the *Tarsl2*-null mouse, the *tarsl2*^−/−^ zebrafish line appeared phenotypically normal. These results suggest that Tarsl2 is not a tRNA synthetase, at least in physiological conditions, since its evolutionary emergence in vertebrates.

## Discussion

Although a chimeric protein from the N-terminal domain of Tars1 and the main body of Tarsl2 catalyzes tRNA^Thr^ aminoacylation and Ser-tRNA^Thr^ editing *in vitro* (22), our genetic data clearly proved that loss of *Tarsl2* did not influence the total or aminoacylated tRNA^Thr^ levels *in vivo*. In addition, *Tars1* is an essential gene despite the emergence of *Tarsl2*. These observations suggest that *Tarsl2* did not evolve for tRNA^Thr^ aminoacylation in physiological conditions. Failure of Tarsl2 in supporting survival at the absence of Tars1 is probably attributable to its very low amount compared to Tars1 and other aaRSs *in vivo* (22, 24). Indeed, the mRNA level of *Tars1* is 3.4- to 100-times higher than that of *Tarsl2* in various mouse tissues (22). MS analyses have also shown that protein abundance of TARSL2 is much lower than that of TARS1 (24). In addition, its nuclear distribution due to an nuclear localization signal (NLS) in the C terminus may also prevent the charge of tRNA^Thr^, whose localization is rather cytoplasmic (22). Despite being bound by Rars1 and p43 (23), loss of Tarsl2 did not influence the incorporation of the two proteins in the MSC, suggesting that Tarsl2 is a peripheral component. The integrity of the MSC remains unchanged, which is consistent with the fact that the amount of tRNA and the charging levels of noncognate tRNAs are not changed. These results suggest that Tarsl2 plays little role in protein synthesis, at least in physiological conditions. We cannot exclude the possibility that, during specific stimuli or in stress conditions, Tarsl2 is able to charge tRNA^Thr^ for protein synthesis. Alternatively, Tarsl2 perhaps participates in other pathways of tRNA metabolism, such as processing or modification.

Although Tarsl2 plays little role in tRNA aminoacylation, it is clearly essential for the growth of mice. Specifically, an elevated metabolism rate was observed after the loss of *Tarsl2*. In line with its high expression in muscle (22), muscle development in *Tarsl2*-KO mice was impaired. Lars1 negatively regulates myogenic differentiation and injury-induced skeletal muscle regeneration mediated by the Rag–mTORC1 pathway (37). Similarly, TARS1 has been recently found to be a key negative regulator in muscle development by inhibiting c-Jun N-terminal Kinase (JNK) signaling. The N-terminal extension of TARS1 has been found to be essential in the regulation of myoblast differentiation (38). Notably, Tarsl2 forms a heterodimer with Tars1 *in vivo* (23) but their N-terminal extensions differ. To understand whether Tarsl2 affects the JNK pathway similar to Tars1 or *via* Tars1, we measured the phosphorylation of JNK after Tarsl2 expression in C2C12 cells and we found that the total and phosphorylated JNK levels were unaltered before and after the induction of muscle differentiation (data not shown). Thus, the precise function of Tarsl2 in muscle development needs further exploration.

In addition to the involvement of TARS1 in muscle development, at least two other noncanonical functions of TARS1 have been described. TARS1 is secreted during inflammation to stimulate endothelial cell migration and angiogenesis (39). Considering the high similarity between TARS1 and TARSL2, TARSL2 may be released from cells, but evidence for this remains to be determined. In addition, TARS1 participates in translational initiation regulation to positively regulate mRNA translation in vertebrates (40). In this context, the N-terminal extension of TARS1 is required for the TARS1 interaction with 4EHP. Considering the completely distinct architectures of the two extensions, the possibility that TARSL2 is involved in mRNA translation initiation is low.

We have recently reported that a nonsense mutation in zebrafish *Tars1*, leading to an inactive truncated Tars1, causes abnormal angiogenesis (36). Obviously, we found that angiogenesis keeps unchanged in the absence of *Tarsl2*, clearly showing that Tarsl2 is not involved in angiogenesis in zebrafish. In addition, the body length of adult zebrafish between WT and *Tarsl2*-deleted species is comparable, in contrast to the obvious growth impairment of *Tarsl2* KO mice. Regarding the seemingly different phenotypes between the mouse and zebrafish models, it has been widely noticed and accepted that many metabolic stress-related phenotypes in zebrafish appear relatively mild compared to those in the mouse (41). Possible mechanistic explanations for this phenomenon may include (1): early development of zebrafish embryos relies more on the maternally deposited mRNAs and proteins, because their zygotic genes are not expressed until 1000-cell stage, equivalent to 2-cell stage of mice (2, 42) zebrafish embryos can obtain nutrients from their yolk, and this process can be a passive diffusion independent of forming a complicated vasculature system (43); and (3) zebrafish can tolerate a higher degree of starvation stress, as it has been shown that zebrafish larvae can live up to 17 days postfertilization without any food supply (44).

*Tarsl2* is the only aaRS duplicated gene in vertebrates. Tarsl2 encompasses a range of functions and roles that include the catalytic functions of aminoacylation and editing (22) and the ability to be incorporated in the MSC (23). Tarsl2 is also essential for growth, metabolism, and muscle development, however, its precise molecular function *in vivo* is still unclear and needs to be further explored. The ability of Tarsl2 to bind amino acid and nucleic acids (22) suggests that it may sense amino acid levels like LARS1 in mTOR signaling pathway (45, 46) or bind RNA or DNA in cytoplasm and/or nucleus, like other tRNA synthetases (47, 48). In addition, with two conserved LZs in the N-terminal extension, Tarsl2 probably interacts with other yet unidentified proteins by hydrophobic interactions.

## Experimental procedures

### Materials

Digoxin probes, Tris-base, NaCl, 20 × saline-sodium citrate buffer, 50 × Denhardt’s solution and fish sperm DNA were purchased from Sangon. KOD-plus mutagenesis kits were obtained from TOYOBO. Prestained molecular protein standards, puromycin, and TRIzol reagent were obtained from Thermo Fisher Scientific. An anti-puromycin antibody (MABE343), nylon membrane and polyvinylidene fluoride (PVDF) membrane were obtained from Millipore. An anti-digoxigenin-alkaline phosphatase (AP) antibody, blocking reagent and CDP-star were obtained from Roche. A PrimeScript RT reagent kit was purchased from TaKaRa. The DNA primers and 2 × T5 Fast qPCR Mix (Probe) were obtained from Tsingke. Anti-TARSL2, anti-RARS1, anti-EPRS1, anti-p43, anti-p38, anti-QARS1, anti-KARS1, and anti-TARS1 antibodies were obtained as described in previous reports (23, 49). HRP-labeled anti-mouse and anti-rabbit secondary antibodies were purchased from Sigma‒Aldrich.

### Structural modeling

Full-length monomeric structure models of TARS1 and TARSL2 were obtained from the AlphaFold Protein Structure Database (30, 31). Two monomeric models were superimposed on the human TARS1 structure (PDB ID 4P3N) to generate TARS1 and TARSL2 dimer models (27). Then, two tRNA molecules were docked onto the dimer models in reference to the *E. coli* ThrRS–tRNA complex structure (PDB ID 1QF6) (26).

### Gel filtration chromatography of cell lysates

Mouse tissue extract was applied to a Superose-6 column for HPLC and eluted at a flow rate of 0.5 ml/min by using a buffer containing 50 mM Tris–HCl (pH 7.5), 50 mM NaCl, 1 mM PMSF, and 1 mM DTT. Fractions were collected for immunoblotting (Western blot).

### Western blot analysis

Different proteins in whole-cell lysates were separated by 10% SDS‒PAGE and then transferred to PVDF membranes. The PVDF membrane was then cropped to blot different proteins in the same lane. After blocking with 5% (w/v) nonfat dried milk, the membranes with targeted proteins were incubated with the corresponding primary antibodies overnight at 4 °C. The membranes were then washed three times using PBS supplemented with 0.05% Tween-20 (PBST) (137 mM NaCl, 2.7 mM KCl, 10 mM Na_2_HPO_4_, 2 mM KH_2_PO_4_, and 0.5‰ Tween-20) and incubated with the corresponding horse radish peroxidase-conjugated secondary antibodies at room temperature for 30 min. After washing three times with PBST, the membranes were treated with a chemiluminescent substrate and imaging was performed using an Amersham imager 680 system (GE).

### Generation of the *Tarsl2*-deletion mice

*Tarsl2*-deletion mice were established using CRISPR/Cas9-mediated gene editing technology by Shanghai Model Organisms. Briefly, two guide RNAs (gRNAs) (5′ACCCTTGGTATCCCGAGAACTGG3′ and 5′AGACAACATTAATTGTATACGGG 3’ (PAM sequences are underlined) were designed to target two introns around exon 3 of the *Tarsl2* transcript (ENSMUST00000032728.8). Cas9 (New England Biolabs, M0646T) and gRNAs (obtained by *in vitro* transcription) were then injected into C57BL/6J mouse zygotes to obtain F0 generation mice, which were subsequently crossed with C57BL/6J mice to obtain F1 generation mice. Gene editing led to the deletion of a gene fragment harboring exon 3 and premature termination of mRNA translation at the Val codon^114^. Different primers (Primers 1 and 2 or Primers 3 and 4, Table S1) were used to identify the *Tarsl2*^+/+^, *Tarsl2*^+/−^, and *Tarsl2*^−/−^ mice.

### Generation of the Tars1 deletion mice

*Tars1*-deletion mice (C57BL/6N-*Tars^em1Cya^*) were purchased from Cyagen Biosciences, which were constructed using CRISPR/Cas9-mediated gene editing technology. Two gRNAs (5′TCTCTTAGGATGCCCTCCTAAGGAGG3′, and 5′GCTTGTGACTGACTAAGATCAGG 3′ (PAM sequences are underlined) were designed to target two introns (located upstream of exon 3 and downstream of exon 11) of the *Tars1* transcript (ENSMUST00000022849). Cas9 (New England Biolabs, M0646T) and gRNAs (obtained by *in vitro* transcription) were then injected into C57BL/6J mouse zygotes to obtain F0 generation mice, which were subsequently crossed with C57BL/6J mice to obtain F1 generation mice.

### Real-time PCR quantification of mRNA

Total RNA was isolated from mouse muscle or zebrafish using TRIzol reagent as described in the Methods. Complementary DNA (cDNA) was synthesized following the procedures of the PrimeScript RT reagent kit protocol. Real-time qPCR was then performed using 2 × T5 Fast qPCR Mix. The sequences for qPCR quantification are listed in Table S1.

### Northern blot analysis

Total RNA extracted from muscle of 6-week-old mice was isolated using TRIzol reagent, and 5 μg of total RNA was electrophoresed through a 15% polyacrylamide-8 M urea gel in Tris-borate-EDTA buffer at room temperature at 150 V for 1.5 h. For aminoacylation assays, total RNA was extracted and resolved with 0.1 M NaAc (pH 5.2). To separate the charged and uncharged tRNAs, 5 μg of total RNA was electrophoresed through an acidic (pH 5.2) 10% polyacrylamide-8 M urea gel at 4 °C with 8 W for 16 h. The RNA was then transferred onto a positively charged nylon membrane at 4 °C under 250 mA for 30 min. After UV-crosslinking (8000 × 100 μJ/cm^2^), the nylon membrane was blocked with prehybridization solution (4 × saline-sodium citrate, 1 M Na_2_HPO_4_, 7% SDS, 1.5 × Denhardt’s solution, 0.4 mg/L fish sperm DNA) at 55 °C for 2 h. The blocking solution was substituted for hybridization solution with digoxin-probes (the probe sequences are listed in Table S2) for specific tRNAs and 5S RNA and incubated at 55 °C overnight. The membrane was washed twice with wash buffer (0.1 M maleic acid and 0.15 M NaCl, pH 7.5) for 15 min each time, and then, 1 × blocking reagent (0.1 M maleic acid, 0.15 M NaCl, and 10% blocking reagent, pH 7.5) was added and incubated for 30 min at room temperature. Next, the cells were incubated with anti-AP buffer (1 × blocked reagent and anti-digoxigenin-AP antibody diluted 1:10,000) for 1 h and washed twice with washing buffer. The membrane was treated with CDP-Star, and imaging was performed using an Amersham imager 680 system (GE).

### Puromycin incorporation assay

Primary mouse embryonic fibroblasts (MEFs) were prepared after isolation from WT and *Tarsl2*^−/−^ mice according to a standard protocol (50) and cultured in Dulbecco's modified Eagle's medium supplemented with 10% fetal bovine serum (FBS). After reaching approximately 95% confluence, fresh Dulbecco's modified Eagle's medium (supplemented with 10% FBS and 1.5 μg/ml puromycin) was used to culture the cells for 2 h. The cells were then collected and lysed, and the newly synthesized proteins with incorporated puromycin were detected with an antipuromycin antibody by Western blotting.

### RNA-sequencing

Total RNA was extracted from muscle in 6-week-old *Tarsl2*^+/+^ and *Tarsl2*^−/−^ mouse and isolated using TRIzol reagent. The RNA-seq libraries were prepared using Illumina TruSeq Stranded mRNA Library Prep Kit Set A (RS-122-2101, Illumina), starting with 2 μg of total RNA as input material. cDNA library preparation and sequencing were performed according to the Illumina’s standard protocol. Library size selection was performed using Agencourt AMPure XP beads (Beckman Coulter, A63882), resulting in an average library size of 400 bp. The libraries were sequenced using Illumina NovaSeq 6000 with paired-end 150 bp reads. Sequencing quality was assessed by FastQC v.0.11.4. All reads were mapped to the reference genome of Illumina iGenomes UCSC mm10 using HISAT2 v.2.05.0. Differential expression analysis was implemented using DESeq2 R package (1.20.0).

### Histology

The 8-week-old *Tarsl2*^+/+^ and *Tarsl2*^−/−^ mouse femurs were fixed in 4% paraformaldehyde for 48 h and specimens were decalcified until soft and pliable. Samples were incubated in 15% diethyl pyrocarbonate–EDTA (pH 7.8) for decalcification. Then, specimens were embedded in paraffin and sectioned at 5 μm. Muscle tissues were embedded with optimal cutting temperature (O.C.T.) Compound (Sakura) and frozen in liquid nitrogen for 1 min. The samples were preserved at −80 °C. Then, the samples were sectioned at 10 μm thickness and stained with H&E.

### Myofiber cross-section area measurement

The cross-sectional area of the myofibers section images were obtained from TA muscles. Three-hundred fibers were chosen randomly from each mouse and were measured. The myofiber cross-section area was measured by Image J software (https://imagej.nih.gov/ij/).

### μCT analysis

The 8-week-old *Tarsl2*^+/+^ and *Tarsl2*^−/−^ mouse femurs were collected. Soft tissues were removed and the remaining tissues were fixed in 70% ethanol. Scanning was performed with the microquantitative computed tomography SkyScan 1176 System. The mouse femurs were scanned at an 8.96 μm resolution for quantitative analysis. The interesting region segmented by a fixed threshold was reconstructed into three-dimensional images. The properties of Tb.Th, Tb.N, BV/TV, C.Th were then quantified.

### Zebrafish strains

*Tübingen* (ZFIN ID: ZDB-GENO-990623-3), transgenic (Tg, *flk1:EGFP*) (ZFIN ID: ZDB-ALT-050916-14) (51) and *tars* heterozygous zebrafish lines (36) were cultured under standard conditions (28.5 °C, 12 h of light and 12 h of dark) in system water. Embryos were grown in egg water (containing 60 μg/ml sea salt and 0.2% methylene blue). To prevent pigmentation, 0.045% N-phenylthiourea (PTU; Sigma‒Aldrich) was used.

### Generation of *tarsl2*-KO zebrafish by the CRISPR/Cas9 system

The zebrafish *tarsl2* gRNA (5′- GAATGGGAGCGATTTCAGG-3′) was designed by Zinc Finger Targeter (ZiFiT) software (http://zifit.partners.org/ZiFiT/CSquare9Nuclease.aspx). gRNA was obtained with an *in vitro* transcription system and then coinjected with 200 ng/ml Cas9 protein (New England Biolabs, M0646T) to generate one-cell stage embryos according to a method described previously (36). The primers for genotyping are listed in Table S1.

### Angiogenic phenotype assay

Live Tg (*flk1:EGFP*) transgenic embryos were anesthetized with 0.03% tricaine (Sigma; A5040) and then mounted on dishes with 1% low-melting agarose (Sangon Biotech; A600015). Fluorescence images were captured under a scanning confocal microscope (Nikon A1). Blood vessels in the region of the trunk encompassing 7-9 intersomitic vessels (ISVs) were analyzed to quantify the angiogenic phenotype. The number of ectopic branching points per ISV (52) was statistically calculated.

### Animal experiments

The animal experiments with zebrafish were approved by the Committee of Animal Use for Research at Shanghai Jiao Tong University School of Medicine. Animal experiments with mice were approved by the Institutional Animal Care and Research Advisory Committee at the Shanghai Institute of Biochemistry and Cell Biology, Chinese Academy of Sciences. All methods were performed in accordance with the relevant guidelines and regulations of the Shanghai Institute of Biochemistry and Cell Biology, Chinese Academy of Sciences.

## Data availability

Raw RNA-seq data were deposited in the NCBI Gene Expression Omnibus database with accession number GSE218318. All of the study data are included in the article and/or supporting information.

## Supporting information

This article contains supporting information.

## Conflict of interests

The authors declare that they have no known competing financial interests or personal relationships that could have appeared to influence the work reported in this paper.
