# Supplementary material for: Loss of threonyl-tRNA synthetase-like protein Tarsl2 has little impact on protein synthesis but affects mouse development
Source: J Biol Chem. 2023 Apr 12;299(5):104704. doi: 10.1016/j.jbc.2023.104704 (PMC10200997; doi:10.1016/j.jbc.2023.104704)
Supplement: Supplemental data [file mmc1.docx]

Supplementary Data


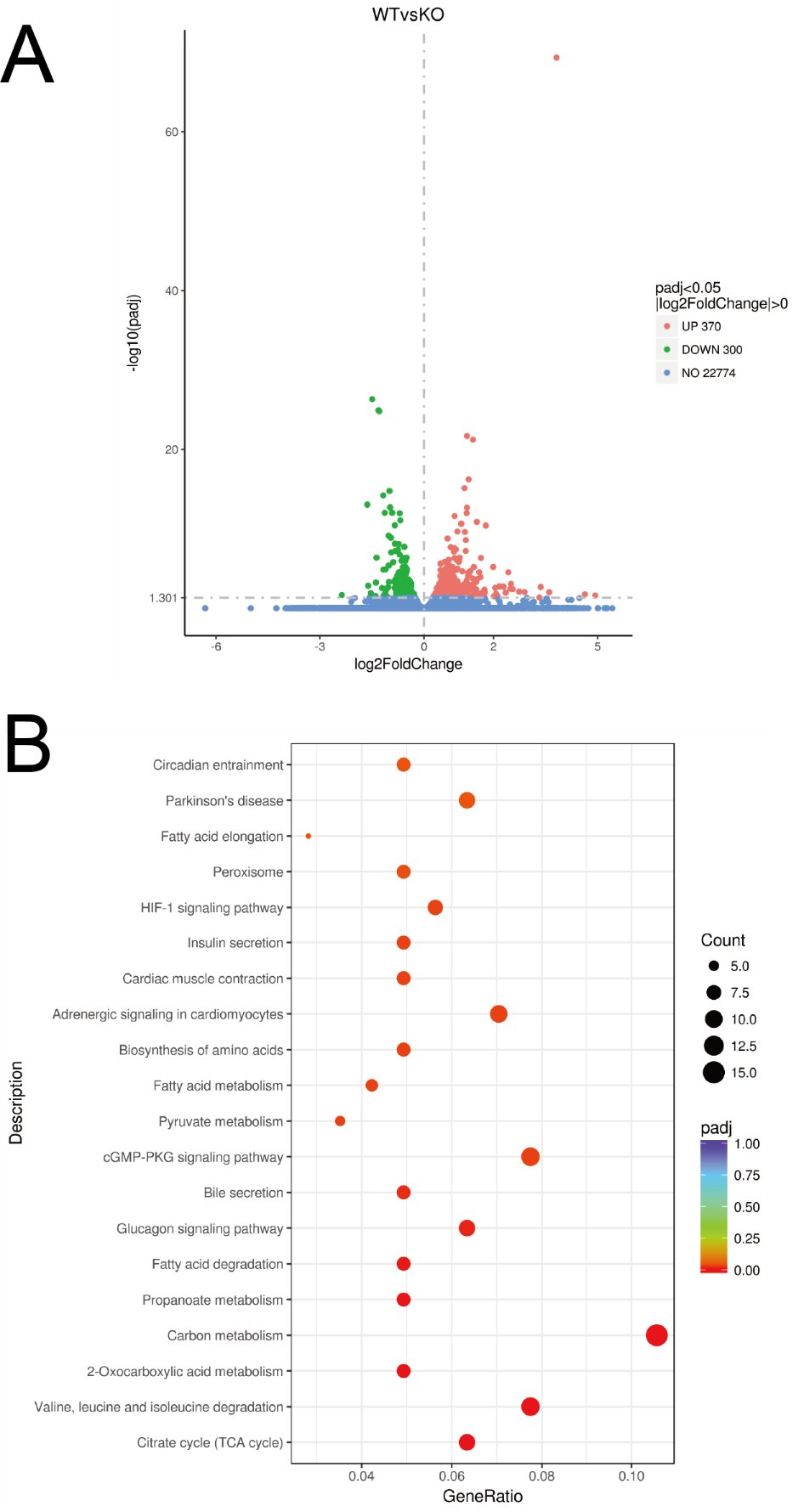


**Supplement Figure 1. Deletion of *Tarsl2* affects the mice metabolism**

(A) Analysis of RNAseq of the *Tarsl2*^+/+^ and *Tarsl2*^-/-^ mice (n=3) muscle results by volcanic map. (B) Analysis of RNAseq of the *Tarsl2*^+/+^ and *Tarsl2*^-/-^ mice muscle results using KEGG pathway enrichment analysis dot graph.


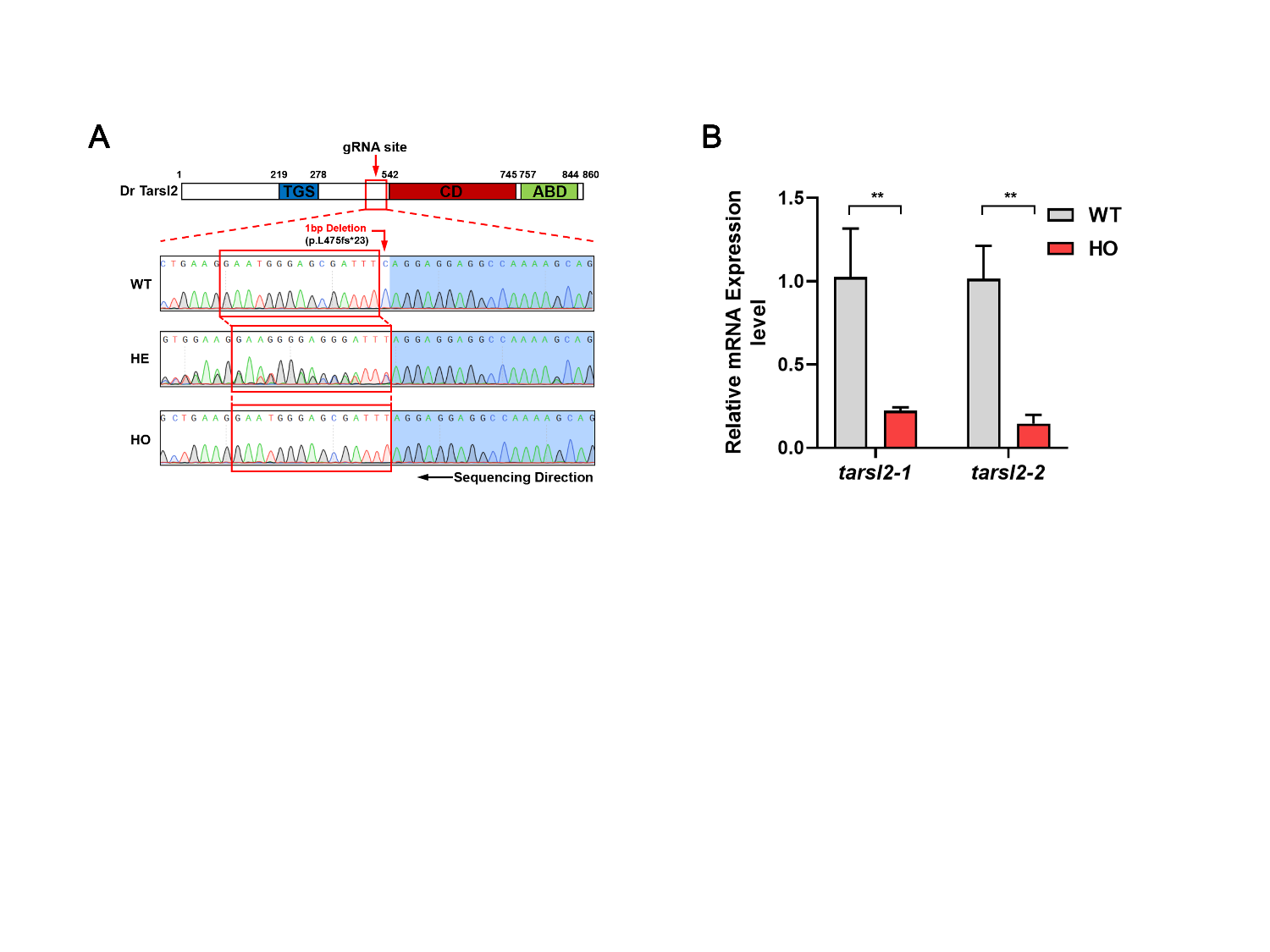


**Supplementary Figure 2. Construct *tarsl2*-null (*tarsl2*^-/-^) zebrafish line.**

**(A)** DNA sequencing results of wild type (WT), *tarsl2* heterozygous (HE, *tarsl2*^+/-^) and *tarsl2* homozygous mutant (HO, *tarsl2*^-/-^) zebrafish. This *tarsl2*^-/-^ zebrafish line contains a 1 bp deletion in *tarsl2* locus, which causes a premature stop codon occurred at codon L475. **(B)** RT-qPCR analysis of *tarsl2* mRNA expression in WT and *tarsl2*^-/-^ embryos using two pairs of primers, respectively, at 5 dpf. Data are presented as means ± SD; two-tailed t-test; **P < 0.01; ***P < 0.001; n.s., not significant.

**Supplementary Table 1. Primers of qPCR and mouse genotyping**

| **Primers for RT-qPCR** | |
| --- | --- |
| **Gene** | **Primer sequence (5’-3’)** |
| ***Tarsl2-1*** | Forward: TCGAGAAGGACACTTTCGCC |
|  | Reverse: CACCATGCAGAAGATGTGCG |
| ***Tarsl2-2*** | Forward: TTCGCCCTCAAACCCATGAA |
|  | Reverse: CGCTTAAAGTGCCTGACAGC |
| ***Gapdh*** | Forward: AGGCCGGTGCTGAGTATGTC |
|  | Reverse: TGCCTGCTTCACCACCTTCT |
| ***Tarsl2*** | Forward: TCGAGAAGGACACTTTCGCC |
|  | Reverse: CACCATGCAGAAGATGTGCG |
| ***Ppara*** | Forward: ACGCATGTGAAGGCTGTAAG |
|  | Reverse: CACTTGTGAAAACGGCAGTAC |
| ***MyHC*** | Forward: CGCAGAATCGCAAGTCAATA |
|  | Reverse: ATATCTTCTGCCCTGCACCA |
| ***MyoG*** | Forward: CGGCTGCCTAAAGTGGAGAT |
|  | Reverse: AGGCCTGTAGGCGCTCAA |
| ***Pdk4*** | Forward: AAGATGCTCTGCGACCAGTAT |
|  | Reverse: GAAGGTGTGAAGGAACGTACA |
| ***Apoc4*** | Forward: GGGACAGATGGCAGTGGTTC |
|  | Reverse: GGCTGTGGGTCTTGTTTAGGA |
| ***Pgc1a*** | Forward: GCAGCCAAGACTCTGTATGG |
|  | Reverse: TTCCGATTGGTCGCTACACC |
| ***Pgc1b*** | Forward: CTCTGACACGCAGGGTGG |
|  | Reverse: GAAGAGCTCGGAGTCATCGG |
| ***Igf2*** | Forward: GCAAACTGGACATTAGCTTCT |
|  | Reverse: ACTGAAGCGTGTCAACAAGCTC |
| ***Zebrafish tarsl2-1*** | Forward: AGTTTCGTGTCTGCTCGTCTGG  Reverse: GCTGAACAGTGATGGATCTCCC |
| ***Zebrafish tarsl2-2*** | Forward: CGGCTCTGAGATGAAGGGTTGT  Reverse: GATGTCGATCTTGGGTCCGTAG |
| ***Zebrafish β-actin*** | Forward: TGCTGTTTTCCCCTCCATTG  Reverse: TTCTGTCCCATGCCAACCA |
| ***tarsl2-genotyping*** | Forward: GGGTCAGTTTGCTGGAGAAC  Reverse: GGATCGCAGGGAAAACTACA |
| **Primers for mouse genotyping** | |
| **Primer 1** | GCACAGGCAGCCCCCAATG |
| **Primer 2** | CTCCAGCAGCCCTTCCCCTCTTAC |
| **Primer 3** | TGGGAGGATGTGAGAAGATGTGCT |
| **Primer 4** | TAGGCCCCTTGTGAAATGTGAGTC |

**Supplementary Table 2. DIG-labeled probes for Northern Blot**

| **RNA** | **Probe sequence (5’-3’)** |
| --- | --- |
| **mctRNA^Thr^(AGU)** | AGGCGCTTTAACCAACTAAGCCACGGCGCC |
| **mctRNA^Thr^(CGU)** | ACCGACGCCTTACCACTTGGCCACCGCGCC |
| **mctRNA^Thr^(UGU)** | CCAGTGCTCTAACCCCTGAGCTATGGAGCC |
| **mctRNA^Lys^(CUU)** | CCCATGCTCTACCGACTGAGCTAGCCGGGC |
| **mctRNA^Leu^(AAG)** | CAGCGCCTTAGACCGCTCGGCCACGCTACC |
| **mctRNA^Gln^(CUG)** | CCAGAGTGCTAACCATTACACCATGGAACC |
| **mctRNA^Arg^(ACG)** | CAGACGCGTTATCCATTGCGCCACTGGCCC |
| **mctRNA^Ile^(AAU)** | CCACGCTCTAACCAACTGAGCTAACCGGCC |
| **mctRNA^Ile^(UAU)** | TGGTGCTCCAGGTGAGGCTTGAACTCACAA |
| **5S RNA** | GGGTGGTATGGCGGTAGAC |
| **Zebrafish-tRNA^Thr^(AGU)** | AGGCGCTTTAACCAACTAAGCCACGGCGCC |
| **Zebrafish-tRNA^Thr^(CGU)** | ACCGACGCCTTACCACTTGGCCACCGCGCC |
| **Zebrafish-tRNA^Thr^(UGU)** | GCTCTAACCACTGAGCTATGGAGCC |
| **Zebrafish-tRNA^Gly^ (GCC)** | CGCGTGGCAGGCGAGAATTC |
| **Zebrafish-5S RNA** | GCAACCTAGTTTTCCCATGTGGTCTCCAT |
